# Risk functions with outcome measurement error

**DOI:** 10.1093/biostatistics/kxaf052

**Published:** 2026-01-20

**Authors:** Jessie K Edwards, Stephen R Cole, Paul N Zivich, Benjamin Ackerman, Sonia Napravnik, Heather Henderson, Timothy Lash, Bonnie E Shook-Sa

**Affiliations:** Department of Epidemiology, University of North Carolina at Chapel Hill, 135 Dauer Dr., 2101 McGavran-Greenberg Hall CB#7435, Chapel Hill, NC 27510, United States; Department of Epidemiology, University of North Carolina at Chapel Hill, 135 Dauer Dr., 2101 McGavran-Greenberg Hall CB#7435, Chapel Hill, NC 27510, United States; Department of Epidemiology, University of North Carolina at Chapel Hill, 135 Dauer Dr., 2101 McGavran-Greenberg Hall CB#7435, Chapel Hill, NC 27510, United States; Johnson and Johnson, 501 George St, New Brunswick, NJ 08901, United States; School of Medicine, University of North Carolina at Chapel Hill, 321 S Columbia St, Chapel Hill, NC 27599, United States; School of Medicine, University of North Carolina at Chapel Hill, 321 S Columbia St, Chapel Hill, NC 27599, United States; Department of Epidemiology, Emory, 1518 Clifton Rd N E, Atlanta, GA 30322, United States; Department of Biostatistics, University of North Carolina at Chapel Hill, 135 Dauer Dr., Chapel Hill, NC 27510, United States

**Keywords:** data linkage, mortality, outcome measurement errors, survival analysis

## Abstract

Mortality risk estimated from studies that ascertain date of death through linkage to vital statistics registries may be subject to outcome measurement error. As a result, some deaths among study participants may not be captured, some study participants who are alive may be falsely categorized as deceased, and some deaths may be recorded at incorrect times, leading to bias in estimates of mortality risk and survival. Here, we illustrate an extension of the Rogan-Gladen estimator to account for outcome measurement error in risk and survival functions in settings with right censoring. As a motivating application, we consider and account for outcome measurement error that could be induced by incomplete and/or incorrect linkage to death registries when estimating mortality risk among people entering care for HIV in the University of North Carolina Center for AIDS Research HIV Clinical Cohort between 2001 and 2022. A series of simulation studies demonstrates that the approach performed well even when participants selected into the validation study were at higher mortality risk than the main study. The proposed approach may be parameterized using internal or external validation data or used as a form of quantitative bias analysis.

## 1. INTRODUCTION

**Fig. 1.**
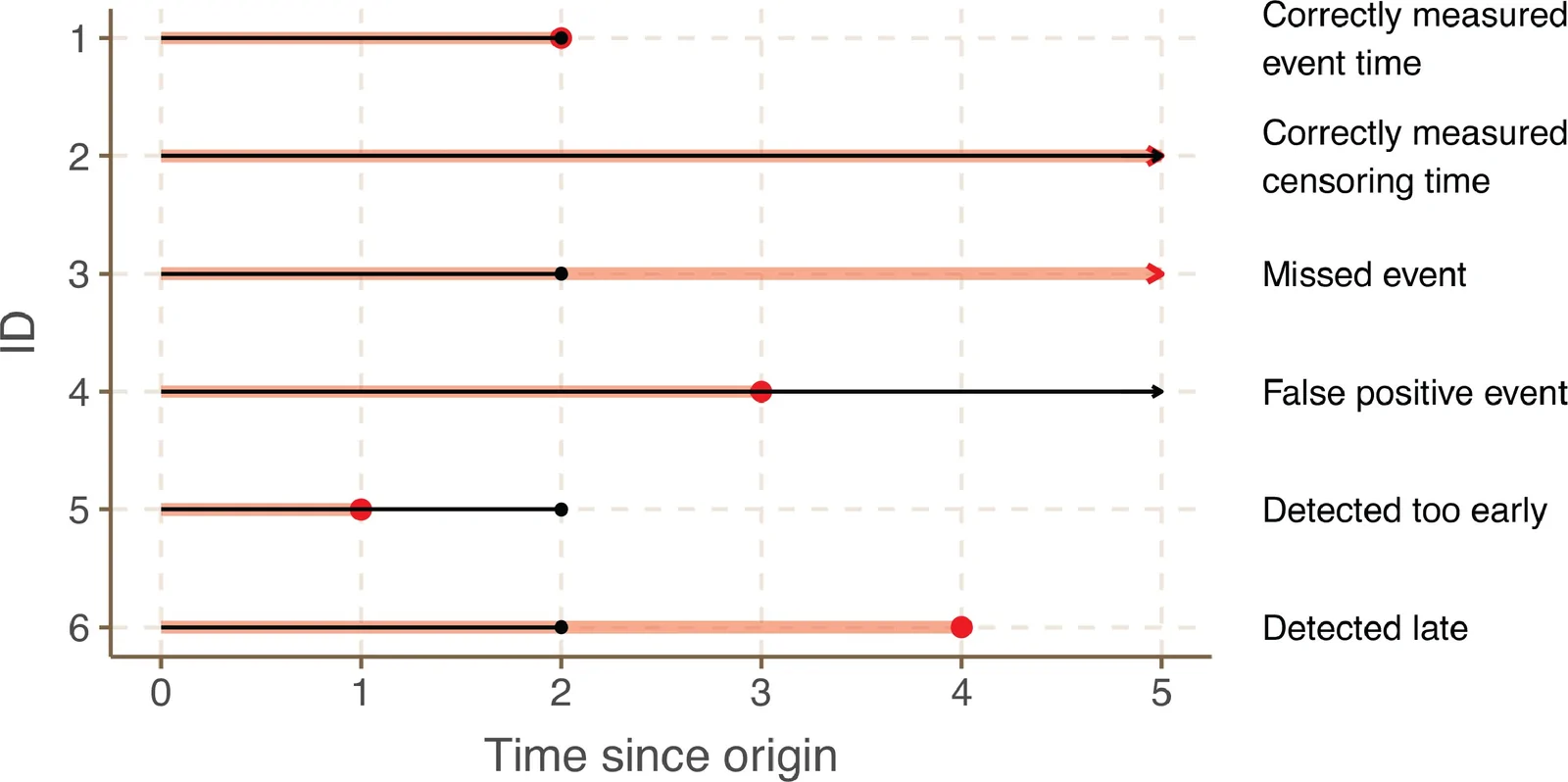
Illustration of types of outcome measurement for time-to-event outcomes.

Risk and survival functions are central measures to summarize incidence of health outcomes in population health research (Benichou and Gail 1990; Cole et al. 2015). Computing a risk or survival function requires information on the event times (Cole and Hudgens 2010). Sometimes these event times are measured correctly (Fig. 1, lines 1 and 2). However, many types of outcomes are subject to measurement error in which events are missed entirely (ie wrongly classified as nonevents at the end of follow-up, line 3), falsely observed (i.e., wrongly classified as events before the end of follow-up, line 4), falsely observed early (ie wrongly classified as events prior to their true event time, line 5), or detected late (ie wrongly classified as nonevents at their event time but classified as events before the end of follow up, line 6), as depicted in Fig. 1. These types of measurement error may occur under many outcome measurement schemes, including settings with self-reported outcome data (eg date of diabetes diagnosis), diagnoses obtained from electronic medical records (e.g., date of myocardial infarction), or outcome data obtained by scheduled testing in a trial or other health research study (e.g., monthly testing for a sexually transmitted infection).

As a motivating application, this work considers estimation of mortality risk in studies that ascertain date of death through linkage to vital statistics registries. In its simplest form, a study might query a death registry by matching on exact name, address, and date of birth. Such linkage is notoriously error prone due to alternative names or spellings, participants changing addresses, and misreporting date of birth on study forms (Brenner et al. 1997). As a result, some deaths among study participants may not be captured and some study participants who are alive may be falsely categorized as deceased, leading to bias in estimates of mortality risk and survival (Oberaigner 2007; Jewell et al. 2020). There is a robust literature on improving protocols linkage to registry data (Shan et al. 2023). Here, we focus on accounting for the measurement error induced by this linkage directly in the risk functions, which is generalizable to other settings with error in the time-to-event distribution. Specifically, we consider and account for possible outcome measurement error when estimating mortality risk among people entering care for HIV in the University of North Carolina Center for AIDS Research HIV Clinical Cohort between 2001 and 2022.

With tabular data derived from a closed cohort, the Rogan-Gladen estimator (Rogan and Gladen 1978) can be used to account for such misclassification when estimating mortality risk. Extensions to this estimator have also been proposed to estimate incidence rates in settings with person-time data under a rare disease assumption (Rothman et al. 2008) and to estimate risk in settings with fixed event screening periods (McKeown and Jewell 2010). Here, we present an approach to account for missed events, false events, and events with mismeasured event times when computing risk or survival functions for which data are subject to right censoring.

Although methods to account for measurement error in binary outcomes are well established, measurement error of time-to-event outcomes has not been widely considered, possibly because several papers have shown that measurement error in the time-to-event produced only small biases in hazard ratios estimated from Cox and Weibull models (Skinner and Humphreys 1999; Korn et al. 2010). However, Skinner and Humphreys (1999) noted that measurement error in times to event did produce bias in the intercept of the Weibull model, implying that such measurement error could produce bias in survival and risk function estimates.

Accurately estimating risk (and survival) functions is an essential task in health research and provides value beyond simple comparisons of hazard functions as given by Cox and accelerated failure time models. Estimating absolute risk of an outcome of interest (eg risk of death after cancer diagnosis) is a fundamental component of descriptive studies and essential for risk communication with patients and their families. Moreover, when addressing causal questions, estimates of risk under each treatment plan or intervention provides better information for decision making than restricting focus to the contrast in risks and circumvents the built-in selection bias of the hazard ratio (Hernán 2010).

Despite these limitations of the hazard ratio, approaches to account for measurement error of event times primarily focus on proportional hazards models and/or rely on discretizing time. Meier et al. (2003) extended the discrete proportional hazards model to account for outcome measurement error with known sensitivity and specificity when estimating survival and the discrete time hazard ratio. Magaret (2008) adapted this approach to incorporate validation data to inform sensitivity and specificity when they are unknown. Giganti et al. (2020) demonstrated use of multiple imputation of outcomes at discrete time points to compute both risks and hazard ratios. Finally, Oh et al. (2018) used SIMEX to account for measurement error of the time to event in the Cox model.

Here, an approach to account for measurement error in event times when estimating risk (or cumulative incidence) functions is proposed. This approach takes as inputs the naive risk function (computed using the possibly mismeasured event time) and functions corresponding to longitudinal extensions of sensitivity and specificity. These functions can be assumed to be known, informed by internal or external validation data, varied across plausible ranges as in sensitivity analyses or quantitative bias analysis, or specified as distributions corresponding to a summary of expert knowledge.

The remainder of this paper is structured as follows. Section 2 details the motivating example. Section 3 presents the notation and approach. Specifically, Section 3.1 presents the estimator, Section 3.2 illustrates how functions for sensitivity and specificity may be estimated nonparametrically or parametrically using validation data, and Section 3.3 demonstrates how these functions may be applied in settings with expert knowledge in place of validation data or as a sensitivity analysis. Section 4 explores the finite sample properties of the proposed approach in a series of simulation experiments. The approach is applied to the motivating example in Section 5, and the paper closes with a discussion in Section 6.

## 2. MOTIVATING EXAMPLE

To motivate this work, we evaluate the risk of mortality among people entering HIV care in the University of North Carolina (UNC) Center for AIDS Research (CFAR) HIV Clinical Cohort (Howe et al. 2010). The UNC CFAR Clinical Cohort enrolls people at least 18 yr old living with HIV seen at the UNC HIV Clinic. Enrollment began in 2001 and has been ongoing through 2025. The cohort follows participants from enrollment through death or disengagement from care at UNC. To ascertain deaths, the cohort regularly queries North Carolina Death Certificate data (MacMahon 1983). In addition to relying on this vital statistics registry, the UNC CFAR Clinical Cohort captures deaths occurring among participants in active HIV care and performs adjudication of all deaths identified through linkage.

When relying on deterministic linkage to state death certificate data, deaths that occur among study participants will not be captured if a participant’s name, date of birth, or other linkage information is recorded differently in the study database and on the death certificate. On the other hand, some participants who are alive could appear to have died due to errors in the linkage process. Both types of outcome measurement error will induce error into the time-to-event distribution used to compute the risk of mortality and survival for this group, which can in turn produce bias in estimates of risk and survival.

While the UNC CFAR Clinical Cohort captures dates of death based on both the gold standard adjudication process and through simple linkage algorithms, full adjudication of death dates is not feasible in many HIV cohort studies. Therefore, the UNC cohort data are used in this application to illustrate the bias arising from simple deterministic linkage algorithms and to allow benchmarking of the proposed approaches to account for measurement error against the gold standard death dates.

## 3. MATERIALS AND METHODS

### 3.1. Parameter and estimators

The parameter of interest is the risk, or * F(t)=P(T≤ t) *, where * T * represents the time from the origin until an event of interest. In the absence of competing events, survival is the complement of risk, such that * S(t)=1-F(t) *. Available data are often subject to right censoring such that we do not observe $ T_{i} $ for all participants. Rather, we observe $ T_{i}^{*}=\mathrm{min}(T_{i},C_{i}) $, where $ C_{i} $ is the censoring time for participant * i *, and the set of event indicators $ \delta_{i}=1 $ if $ T_{i}^{*}=T_{i} $ and $ \delta_{i}=0 $ otherwise. In the presence of right censoring (but no measurement error), risk and survival may be consistently estimated nonparametrically using the Kaplan-Meier estimator (Kaplan and Meier 1958) or the Fleming-Harrington estimator (Nelson 1969; Aalen 1976).

In some cases, a mismeasured version of * T *, which we will label * W *, is subject to right censoring. The Kaplan-Meier and Fleming-Harrington estimators are biased in the presence of such measurement error. Here, we address 2 types of outcome measurement error. First, we consider a nonzero probability of failing to observe an event that occurred prior to time * t *, such that, among those with $ T_{i}\leq t $, at least some participants have $ W_{i} > t $. We will represent the probability of being identified as an event by time * t *, given that one truly had the event at or before * t *, as $ a(t)=P(W\leq t|T\leq t) $.

Second, we consider settings with false positive events that appear to occur before the true event times, such that $ W_{i} < T_{i} $ for some participants * i *. Such false positive events may or may not change the event indicator. For example, some false positive events occur among participants who later had the event such that the event indicator is correct, but the time is too early, while other false positives occur among participants who did not have the event before censoring such that the observed time and the event indicator are both incorrect. We will represent the probability of appearing as a false positive event at or before time * t * as $ b(t)=P(W\leq t|T\,\gt\,t) $.

When * W * is subject to right censoring, we observe $ W^{*}_{i}=\mathrm{min}(W_{i},C_{i}) $ and the event indicator $ \eta_{i}=1 $ if $ W_{i}^{*}=W_{i} $ and $ \eta_{i}=0 $ otherwise. If either * a(t) < 1 * or * b(t) > 0 *, standard estimators of risk at time * t *, which ignore outcome measurement error, may be biased.

To account for this bias, risk at time * t * is estimated by


(1) \begin{align*}\hat{F}(t)=\mathrm{sup}\Bigl\{\frac{\hat{F}_{W}(v)-b(v)}{a(v)-b(v)}; 0\leq v\leq t\Bigr\},\end{align*}

where $ \hat{F}_{W}(t) $ is the Kaplan-Meier estimator of the risk function assuming no measurement error in the observed event times (ie for * W * rather than * T *). Note that the supremum in Equation (1) ensures that $ \hat{F}(t) $ is monotonically increasing. This estimator is a generalization of the Rogan-Gladen estimator to time-to-event data. Derivation of this estimator is provided in the Supplementary Material.

### 3.2. Parameterization using validation data

The estimator above assumes that the functions * a(t) * and * b(t) * are known. However, these complex functions are rarely known *a priori*. If * a(t) * and * b(t) * are unknown, they may be estimated directly from validation data when available. Such validation data would include both the gold standard event times * T * and the possibly mismeasured event times * W * for either a subset of the main study data or for an external study sample.

#### 3.2.1. Nonparametric estimation of * a(t) * and * b(t) * using validation data

In settings with large validation datasets in which $ T^{*} $ and $ W^{*} $ are both observed, * a(t) * and * b(t) * may be estimated nonparametrically. Because * a(t) * and * b(t) * are applied to the observed risk function $ \hat{F}_{W}(t) $, estimates of these parameters are needed at each observed event time. Let * R * represent the vector of ranked (ordered) observed event time, and $ R_{k} $ represent the * kth * observed event time. With sufficiently large validation data, a consistent estimator of $ a(R_{k}) $ is the nonparametric estimator


(2) \begin{align*}\hat{a}_{np}(R_{k})=\frac{\sum_{i=1}^{m}I(W^{*}_{i}\leq R_{k},T^{*}_{i}\leq R_ {k})\delta_{i}\eta_{i}}{\sum_{i=1}^{m}I(T^{*}_{i}\leq R_{k})\delta_{i}}\end{align*}

and a consistent estimator of $ b(R_{k}) $ is the nonparametric estimator


(3) \begin{align*}\hat{b}_{np}(R_{k})=\frac{\sum_{i=1}^{m}I(W^{*}_{i}\leq R_{k},T^{*}_{i} > R_{k})\eta_{i}}{\sum_{i=1}^{m}I(T^{*}_{i} > R_{k})}\end{align*}

where * m * is the number of participants in the validation study. Point-wise consistency proofs are provided in the Supplementary Material.

#### 3.2.2. Parametric estimation of * a(t) * and * b(t) * using validation data

When the validation data contains few events, either because the sample size of the validation study is small or the outcome is rare, nonparametric estimators of * a(t) * and * b(t) * may be unstable due to sparse data. In this setting, parametric estimators of * a(t) * and * b(t) * allow computation of $ \hat{F}(t) $ under a set of parametric assumptions.

First, consider $ b(t)=P(W\leq t|T\,\gt\,t)=F_{W}(t|T\,\gt\,t) $. Rather than estimate * b(t) * nonparametrically, a parametric distribution for * b * could be assumed. For example, if the time to a false positive event is assumed to follow an exponential distribution, or the false positive event rate $ \lambda_{fp} $ is assumed to be constant over time, a parametric estimator for * b * at each time $ R_{k} $ is


(4) \begin{align*}\hat{b}_{p}(R_{k})=1-\mathrm{exp}(-\hat{\lambda}_{fp}R_{k})\end{align*}

where $ \hat{\lambda}_{fp} $ is an estimator of the false positive event rate from the validation data (eg number of false positive events divided by person time at risk for a false positive event).

Recall that $ a(t)=P(W\leq t|T\leq t) $. Developing a parametric estimator for * a(t) * requires considering the 3 processes by which the event might be observed at or prior to * t * given that the true event occurs by that time: events occurring at their true event time (Fig. 1 line 1); events detected early (Fig. 1, line 4); and events detected late (Fig. 1, line 5). Let * a(t)=x(t)+y(t)+z(t) *, where $ x(t)=P(T-\epsilon < W\,\lt\,T+\epsilon|T\leq t) $ for arbitrarily small * ε *, $ y(t)=P(W\,\lt\,T-\epsilon|T\leq t) $, and $ z(t)=P(T+\epsilon < W\leq t|T\leq t) $. In settings with no outcome measurement error, * x(t)=1, y(t)=0 *, and * z(t)=0 *, so that $ a(t)=1\;\forall\; t $.

The quantity * x(t) * is the probability of observing an event within an arbitrarily small window of its true event time, $ T\pm\epsilon $. To connect this quantity more firmly with the definition of sensitivity, * x(t) * is rewritten as


(5) \begin{align*}\begin{array}{ll}x(t)=P(T-\epsilon < W < T+\epsilon|T\leq t)\\=P(T-\epsilon < W < T+\epsilon|T\leq t, W\geq T-\epsilon)P(W\geq T-\epsilon|T\leq t)\\=\theta(t)[1-y(t)]\end{array}\end{align*}

where * θ(t) * corresponds to the probability of detecting a true event within a small window around * T * given that a false positive event has not yet occurred. * θ(t) * may be estimated from the validation data as the number of events detected at their true event time prior to $ R_{k} $ divided by the number of participants at risk at $ R_{k} $ who had not yet had a false positive event.

The quantity * y(t) * is the probability of having a false positive event occur prior to one’s true event, given that the true event happened at or before * t *. Therefore, $ y(t)=F_{W}(T-\epsilon|T\leq t) $. Because * T * is unknown, we approximate * y(t) * by assuming all values of * T * between 0 and * t * are equally plausible and setting * T * midway between time 0 and * t *, or at timepoint * t/2 *. Again assuming the false positive rate is constant over time, $ \hat{y}(R_{k})=1-\mathrm{exp}(-\hat{\lambda}_{fp}[R_{k}/2]) $.

Finally, * z(k) * is the probability of detecting an event after * T * but before * t *. To make a parametric estimator for * z(t) *, * z(t) * is rewritten in terms of the event detection rate as


(6) \begin{align*}\begin{array}{ll} z(t) & =P(T+\epsilon < W\leq t|T\leq t)\\& =P(W\leq t|T\leq t, W > T+\epsilon)P(W > T+\epsilon|T\leq t)\\& =F_{D}(t-T-\epsilon)P(W > T+\epsilon|T\leq t)\\& =F_{D}(t-T-\epsilon)[1-\{y(t)+x(t)\}]\end{array}\end{align*}

where $ F_{D}(t-T-\epsilon) $ is the cumulative probability of event detection by * t-T-ε * time units after the true event time. Again, because * T * is unknown, it is assumed to occur midway between 0 and $ R_{k} $ such that


\begin{align*}\hat{z}(t)=[1-\mathrm{exp}(-\hat{\lambda}_{D}R_{k}/2)][1-\{\hat{y}(t)+\hat{x}(t)\}]\end{align*}

where $ \hat{\lambda}_{D} $ may be estimated from the validation data as the event detection rate (number of late event detections divided by person time between true events and their detection).

Thus, * a(t) * and * b(t) * may be specified for all observed event times by estimating only 3 parameters from the validation data: $ \lambda_{fp} $, * θ(t) *, and $ \lambda_{D} $.

#### 3.2.3. Variance estimation when using validation data

The variance of $ \hat{F}(t) $ may be estimated using the nonparametric bootstrap in which both the main study and validation data are resampled (Greenland 2009). Specifically, in settings with external validation data, * n * participants are resampled from the main study data and * m * participants are resampled from the validation data with replacement in each bootstrap sample * k *. In settings with internal validation data, the bootstrap for the main study data is stratified by inclusion in the internal validation dataset. Then, either $ \hat{a}^{(k)}_{np}(t) $ and $ \hat{b}^{(k)}_{np}(t) $ or $ \hat{a}^{(k)}_{p}(t) $ and $ \hat{b}^{(k)}_{p}(t) $ are computed using Equations (2) and (3) in the sample of the validation data in iteration * k *. Finally, we substitute estimated values of * a(t) * and * b(t) * into Equation (1) to compute $ \hat{F}^{(k)}(t) $. The standard deviation of $ \hat{F}^{(k)}(t) $ across the * k * samples is the estimated standard error and can be used to construct point-wise Wald-type 95% confidence intervals.

### 3.3. Sensitivity analyses using expert knowledge

In some settings, validation data are not available but expert knowledge about $ \lambda_{fp} $, $ \lambda_{D} $, and * θ * may exist. In these settings, the analyst could plug these values into the expressions shown in Section 3.2.2 to compute $ \hat{a}_{p}(t) $ and $ \hat{b}_{p}(t) $ in sensitivity analyses. However, such knowledge is usually subject to a degree of uncertainty, bounded on the lower end by the size of a hypothetical validation dataset that could have given rise to the expert’s opinions about these parameters.

To incorporate this uncertainty into the variance for the parameter of interest, parametric distributions (eg Beta distributions) can be specified for * θ *, * G(τ) * (the cumulative probability of being a false positive by the end of the study), and * Q(s) * (the probability of detecting an event not detected at the time it occurred within * s * days of the true event time, where * s * is user defined).

In each bootstrap sample * k *, the main study data is resampled with replacement, and draws are taken from each of these Beta distributions. From those draws, the false positive rate $ \lambda_{fp} $ and event detection rate $ \lambda_{D} $ can be back calculated based on the exponential distribution under an assumption of constant hazards of false positive events and true event detections. For example, if an investigator believes that 20% of events that are missed on their actual event date are detected within 10 days (* Q(10)=0.2 *), and that this knowledge was based on experience with 100 individuals, they could specify a Beta distribution centered at this value, eg $ Q(10)\sim\mathrm{Beta}(20,80) $. In each draw from this distribution, $ \lambda^{k}_{D} $ would be $ -log(1-Q(10)^{(k)})/10 $.

In practice, uncertainty may be greater than this binomial error and distributions around the values of $ \lambda_{fp} $, $ \lambda_{D} $, and * θ * could be inflated or constructed using alternative means to capture this uncertainty. The relative merits of various systems of eliciting expert knowledge of statistical distributions have been described by Johnson et al. (2010), Falconer et al. (2022), and O’Hagan (2019). An example pertaining to outcome measurement error has been described by Goldsmith et al. (2023).

Regardless of how distributions for * θ *, * G(τ) * and * Q(s) * are constructed, the back calculations described above can be used to compute $ \hat{a}^{(k)}_{p}(t) $, $ \hat{b}^{(k)}_{p}(t) $, and $ \hat{F}^{(k)}(t) $. The 2.5th and 97.5th percentiles of $ \hat{F}^{(k)}(t) $ are taken as the 95% confidence limits.

In the case where knowledge about the validity of the outcome measure is scarce, the approach described above could be applied at various values corresponding to plausible degrees of outcome measurement error to explore the sensitivity of study results to reasonable departures from the assumption of perfect outcome measurement. This process could also be used as part of a larger quantitative bias analysis (Fox et al. 2021).

## 4. SIMULATIONS

### 4.1. Data generating mechanism

To explore the finite sample properties of the proposed approach, we generated 10,000 cohorts each of size $ n\,{=}\,5,000 $. In each cohort, the true time to event * T * followed a Weibull distribution such that $ F_{T}(t)=1-\mathrm{exp}[(-\lambda_{T}t)^{\alpha}] $, where $ \alpha\,{=}\,2 $ and $ \lambda_{T} $ was selected to yield 20% events at 2 yr: $ F_{T}(2)=0.2 $, ($ \lambda_{T}=0.236 $). Note that $ \alpha\,{=}\,2 $ implies that the hazard function is increasing over time. To illustrate issues that could arise from right censoring, we set the probability of being censored before the end of follow-up at 2 yr to 50% in each simulated cohort; among those censored before the end of follow-up, the censoring time * C * was a uniform random variable between 0 and 2. Simulated individuals who remained uncensored and event free at 2 yr were censored at 2 yr.

To explore the impact of validation study sample size on the performance of the estimators, the simulation experiments were performed with validation study sample sizes ranging from $ m\,{=}\,50 $ and $ m\,{=}\,5,000 $. For each sample size, 10,000 validation samples were generated. In each simulated validation sample, the true time to event followed a Weibull distribution with parameters selected such that the shape of the hazard function was the same as in the main study, but the overall risk was higher. This elevated risk in the validation study reflects settings where inclusion in the validation study is associated with predictors of the outcome. Specifically, $ F_{T_{V}}(t)=1-\mathrm{exp}[(-\lambda_{T_{V}}t)^{\alpha}] $, where $ \alpha\,{=}\,2 $ and $ \lambda_{T_{V}} $ was selected to yield 35% events at 2 yr ($ \lambda_{T_{V}}=0.328 $).

Each of the * 10,000 * cohorts was then subjected to 6 measurement error scenarios. We induced measurement error by specifying the probability of detecting an event at its true time (given a false positive event had not yet occurred) (* θ *), the false positive rate $ \lambda_{fp} $, and the event detection rate $ \lambda_{D} $ according to the algorithm described below and in Table S1:

1. Scenario A: $ \theta=1 , \lambda_{fp}=0 , \lambda_{D}=0 $, corresponds to IDs 1 and 2 from Fig. 1
2. Scenario B: $ \theta=0.7 , \lambda_{fp}=0 , \lambda_{D}=0 $, corresponds to IDs 1, 2, and 3 from Fig. 1
3. Scenario C: $ \theta=0.7 , \lambda_{fp}=0 , \lambda_{D}=0.3 $, corresponds to IDs 1, 2, 3, and 6 from Fig. 1
4. Scenario D: $ \theta=1 , \lambda_{fp}=0.1 , \lambda_{D}=0 $, corresponds to IDs 1, 2, 4, and 5 from Fig. 1
5. Scenario E: $ \theta=0.7 , \lambda_{fp}=0.1 , \lambda_{D}=0 $, corresponds to IDs 1, 2, 3, 4, and 5 from Fig. 1
6. Scenario F: $ \theta=0.7 , \lambda_{fp}=0.1 , \lambda_{D}=0.3 $, corresponds to IDs 1–6 from Fig. 1

The approach used to simulate the outcome measurement error reported above is described in the Supplementary Material.

To examine the sensitivity of each approach to the size of the validation dataset, the simulations described above were repeated for validation sample sizes ranging from 50 to 5,000. To examine sensitivity of the approach to the shape of the hazard function in the validation dataset, we repeated the simulations with validation studies in which the hazard function was decreasing over time with $ \alpha\,{=}\,0.5 $. Finally, to assess the validity of the approach when the gold standard validation data were themselves mismeasured, we repeated the simulations with validation data in which the gold standard measurements were error prone with $ \theta\,{=}\,0.95 $, $ \lambda_{fp}=0.005 $, and $ \lambda_{d}=0.2 $.

### 4.2. Analysis of simulated data

Four estimators were used to compute risk in each simulated dataset. First, $ \hat{F}_{W}(t) $ was used to estimate risk while ignoring the potential outcome measurement error. Specifically, $ \hat{F}_{W}(t) $ was computed using the Kaplan-Meier estimator of the risk function based on the error-prone event times $ W^{*} $ and censoring indicator * η * in the main study data alone. Next, risk was estimated using the true event times * T * and event indicators * δ * in the validation study alone $ \hat{F}_{T_{V}}(t) $.

To account for outcome measurement error, both parametric and nonparametric forms of the proposed estimator were applied. Specifically, $ \hat{F}_{p}(t)=F(t;\hat{a}_{p},\hat{b}_{p}) $ was the parametric form of the estimator, which used estimates $ \hat{a}_{p} $ and $ \hat{b}_{p} $ obtained by computing $ \hat{\theta} $, $ \hat{\lambda}_{fp} $, and $ \hat{\lambda}_{D} $ in the validation sample and substituting those estimates into the expressions for * a(t) * and * b(t) * in Section 3.2.2. $ \hat{F}_{np}(t)=F(t;\hat{a}_{np},\hat{b}_{np}) $ was the nonparametric form of the estimator, where $ \hat{a}_{np} $ and $ \hat{b}_{np} $ were estimated using Equations (2) and (3), respectively. In some simulated validation studies, $ \hat{a}_{np} $ and $ \hat{b}_{np} $ were undefined at some time points (specifically, timepoints before any events had occurred). If $ \hat{a}_{np} $ was undefined, it was set to 1 and if $ \hat{b}_{np} $ was undefined it was set to 0. When computing both $ \hat{F}_{np}(t) $ and $ \hat{F}_{p}(t) $, the denominator of Equation (1) was * ≤ 0 * for some timepoints in some iterations of the simulation. For these timepoints, $ \hat{F}_{p}(t) $ and $ \hat{F}_{np}(t) $ were set to $ \hat{F}_{W}(t) $.

For each scenario A-F, bias and relative bias in estimates $ \hat{F}_{W}(t) $, $ \hat{F}_{T_{V}}(t) $, $ \hat{F}_{np}(t) $ and $ \hat{F}_{p}(t) $ were summarized at 8 timepoints over follow-up using boxplots, where bias was defined as 100 times the mean difference between the true and estimated risk and relative bias was bias divided by the true risk at that timepoint. Bias and relative bias in the 2-yr restricted mean survival time were also compared. At the end of follow up ($ t\,{=}\,2 $), empirical standard error (ESE), defined as the standard deviation of the bias across all iterations and average standard error (ASE) were compared across estimators. In addition, root mean squared error, defined as $ \sqrt{bias^{2}+ESE^{2}} $, was compared to assess the tradeoff between bias and precision. To assess the validity of the variance estimator, the standard error ratio (SER), defined as ASE/ESE, and 95% confidence interval coverage were compared.

R code to generate and analyze the simulated data can be found at our GitHub page https://github.com/edwardsjk/risk_outcome_measerr.

### 4.3. Simulation results

In the scenario with no measurement error (Scenario A), $ \hat{F}_{W}(t) $, $ \hat{F}_{np}(t) $ and $ \hat{F}_{p}(t) $ produced results with little bias and appropriate 95% confidence interval coverage. As expected, the validation data only estimator $ \hat{F}_{T_{V}}(t) $ produced results with substantial bias even in the setting with no outcome measurement error because the validation study was known to be enriched for participants at higher risk of the outcome. When the validation study was small, $ \hat{F}_{T_{V}}(t) $ was less precise (Table 1).

**Table 1.** Performance of naive and proposed estimators to compute risk at the end of follow up under 2 validation study sample sizes ($ m\,{=}\,200 $ and $ m\,{=}\,2,500 $) with a total study size of $ N\,{=}\,5,000 $.

					$ m\,{=}\,200 $						$ m\,{=}\,2,500 $					
	$ \lambda_{fp} $	$ \lambda_{D} $	* θ *	Estimator	Bias^a^	ESE	RMSE	ASE	SER	Cov	Bias^b^	ESE	RMSE	ASE	SER	Cov
A	0	0	1	$ \hat{F}_{W}(t) $	0	0.7	0.7	0.7	1	0.95	0	0.7	0.7	0.7	1	0.95
A	0	0	1	$ \hat{F}_{T_{V}}(t) $	15	4.2	15.6	4.2	1	0.04	15	1.2	15	1.2	1	0
A	0	0	1	$ \hat{F}_{np}(t) $	0	0.7	0.7	0.7	1	0.95	0	0.7	0.7	0.7	1	0.95
A	0	0	1	$ \hat{F}_{p}(t) $	0	0.7	0.7	0.7	1	0.95	0	0.7	0.7	0.7	1	0.95
B	0	0	0.7	$ \hat{F}_{W}(t) $	−6	0.6	6	0.6	1	0	−6	0.6	6	0.6	1	0
B	0	0	0.7	$ \hat{F}_{T_{V}}(t) $	15	4.2	15.6	4.2	1	0.04	15	1.2	15.1	1.2	1	0
B	0	0	0.7	$ \hat{F}_{np}(t) $	0.2	2.3	2.3	2.5	1.1	0.95	0	1	1	1	1	0.95
B	0	0	0.7	$ \hat{F}_{p}(t) $	0.2	2.3	2.3	2.3	1	0.94	0	1	1	1	1	0.95
C	0	0.3	0.7	$ \hat{F}_{W}(t) $	−5.3	0.6	5.4	0.6	1	0	−5.3	0.6	5.4	0.6	1	0
C	0	0.3	0.7	$ \hat{F}_{T_{V}}(t) $	15	4.3	15.6	4.2	1	0.04	15	1.2	15.1	1.2	1	0
C	0	0.3	0.7	$ \hat{F}_{np}(t) $	0.2	2.1	2.1	2.3	1.1	0.95	0	1	1	1	1	0.95
C	0	0.3	0.7	$ \hat{F}_{p}(t) $	−0.2	2	2	2.1	1	0.92	−0.4	1	1	1	1	0.92
D	0.1	0	1	$ \hat{F}_{W}(t) $	8	0.8	8	0.8	1	0	8	0.8	8	0.8	1	0
D	0.1	0	1	$ \hat{F}_{T_{V}}(t) $	14.9	4.2	15.5	4.2	1	0.04	15	1.2	15	1.2	1	0
D	0.1	0	1	$ \hat{F}_{np}(t) $	0.2	3.3	3.3	3	0.9	0.91	0	1.3	1.3	1.3	1	0.95
D	0.1	0	1	$ \hat{F}_{p}(t) $	0	2.4	2.4	2.5	1	0.94	0	1.1	1.1	1.1	1	0.95
E	0.1	0	0.7	$ \hat{F}_{W}(t) $	2.4	0.7	2.5	0.7	1	0.08	2.4	0.7	2.5	0.7	1	0.08
E	0.1	0	0.7	$ \hat{F}_{T_{V}}(t) $	15	4.2	15.6	4.2	1	0.04	15	1.2	15.1	1.2	1	0
E	0.1	0	0.7	$ \hat{F}_{np}(t) $	3.3	9.7	10.3	11.2	1.2	0.96	2.0	7.3	7.6	5.4	0.7	0.97
E	0.1	0	0.7	$ \hat{F}_{p}(t) $	0.4	4.3	4.4	4.5	1	0.97	0.2	1.7	1.7	1.6	0.9	0.95
F	0.1	0.3	0.7	$ \hat{F}_{W}(t) $	3.1	0.7	3.2	0.7	1	0.01	3.1	0.7	3.1	0.7	1	0.01
F	0.1	0.3	0.7	$ \hat{F}_{T_{V}}(t) $	15.1	4.2	15.7	4.2	1	0.04	15	1.2	15.1	1.2	1	0
F	0.1	0.3	0.7	$ \hat{F}_{np}(t) $	3.5	9.8	10.4	9.9	1	0.95	2.1	7.3	7.6	5.4	0.7	0.97
F	0.1	0.3	0.7	$ \hat{F}_{p}(t) $	−0.3	4.1	4.1	4.2	1	0.96	−0.4	1.5	1.6	1.5	1	0.93

$ \hat{F}_{W}(t) $ : naive estimator; $ \hat{F}_{T_{V}}(t) $: estimator limited to validation data; $ \hat{F}_{np}(t) $: nonparametric estimator; $ \hat{F}_{p}(t) $: parametric estimator.

^a^ Monte Carlo standard errors for bias with $ m\,{=}\,200 $ ranged from 0.007 to 0.098.

^b^ Monte Carlo standard errors for bias with $ m\,{=}\,2,500 $ ranged from 0.007 to 0.073.

The naive estimator $ \hat{F}_{W}(t) $ had bias in all scenarios with outcome measurement error, with bias increasing over the follow up period and ranging from −6 to 8 percentage points at the end of follow up (Fig. S1A and B and Fig. S2). The naive approach also produced substantial bias in the 2-yr restricted mean survival time (Fig. S2C and D). When the validation study was large, $ \hat{F}_{np}(t) $ produced results with little bias (* ≤ * 2.1 percentage points bias in risk at the end of follow up and * ≤ * 11 days bias in the restricted mean survival time) in all scenarios, and bias for $ \hat{F}_{np}(t) $ was always less than bias in $ \hat{F}_{W}(t) $. However, even with a large validation dataset, $ \hat{F}_{np}(t) $ was imprecise in scenarios E and F, such that RMSE was greater for $ \hat{F}_{np}(t) $ then for $ \hat{F}_{W}(t) $ in these scenarios. When the validation study was small, $ \hat{F}_{np}(t) $ had noticeable bias at the end of follow up (* ≈ 3 * percentage points) in scenarios E and F, which exceeded bias in the naive estimator.

The parametric estimator $ \hat{F}_{p}(t) $ had little bias (< 0.5 percentage points) in all scenarios at the end of follow up, and bias was similar between scenarios with large and small validation studies. $ \hat{F}_{p}(t) $ was less precise than the naive estimator, but RMSE was always smaller for $ \hat{F}_{p}(t) $ than for $ \hat{F}_{W}(t) $ when the validation study was large. When the validation study was small, precision in $ \hat{F}_{p}(t) $ was reduced such that RMSE was greater for this estimator than for the naive estimator in scenarios E and F. Patterns in bias and relative bias were similar across all time points considered (Figs S2 through S5).

**Fig. 2.**
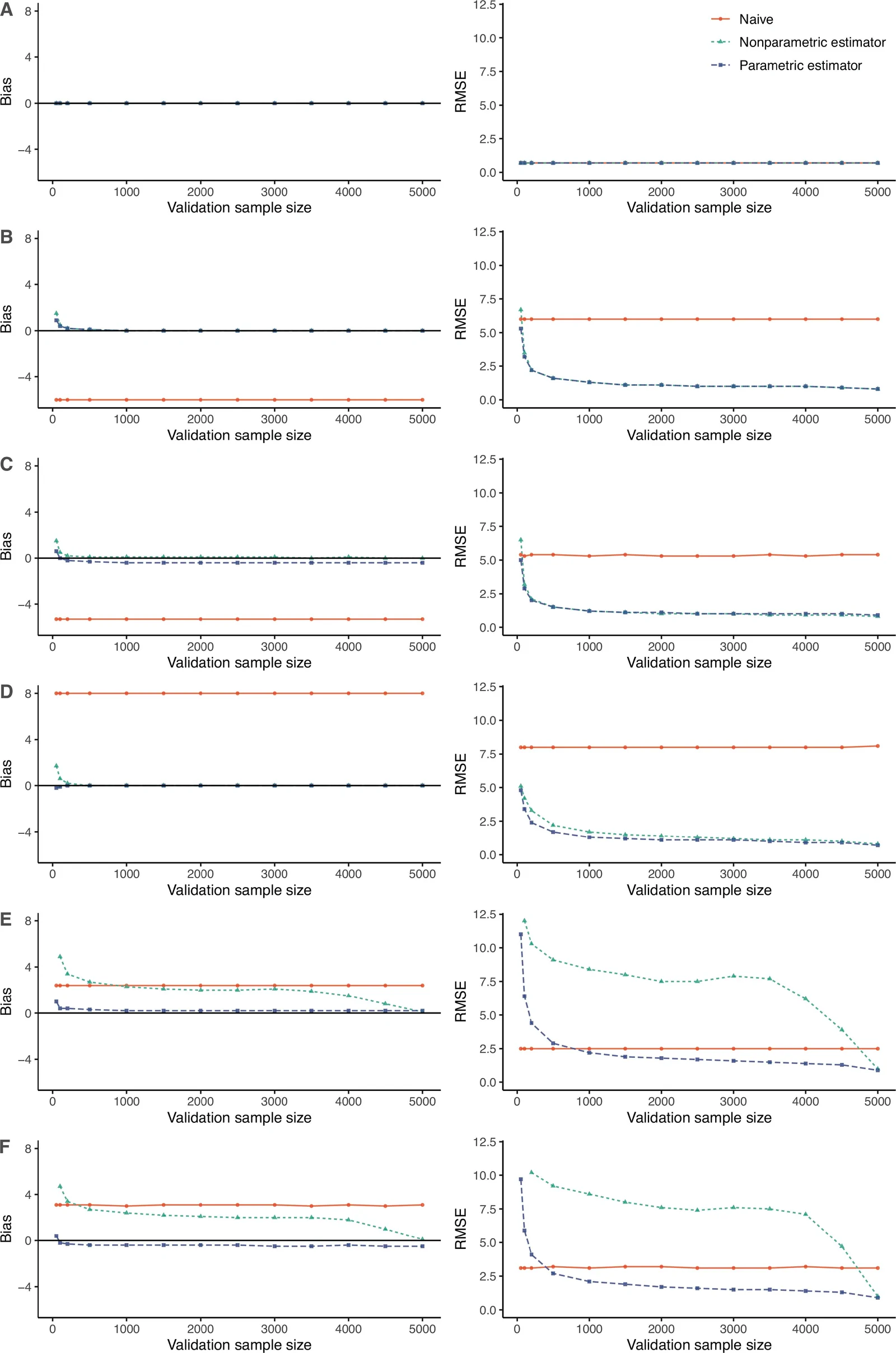
Bias and root mean squared error (RMSE) for the naive estimator $ \hat{F}_{W}(t) $, the nonparametric estimator $ \hat{F}_{np}(t) $, and the parametric estimator $ \hat{F}_{p}(t) $ fit with varying amounts of validation data under 6 measurement error scenarios: A) no measurement error; B) $ \lambda_{fp}=0, \lambda_{D}=0, \theta\,{=}\,0.7 $; C) $ \lambda_{fp}=0;\lambda_{D}=0.3;\theta\,{=}\,0.7 $; D) $ \lambda_{fp}=0.1;\lambda_{D}=0;\theta\,{=}\,1 $; E) $ \lambda_{fp}=0.1;\lambda_{D}=0;\theta\,{=}\,0.7 $; and F) $ \lambda_{fp}=0.1;\lambda_{D}=0.3;\theta\,{=}\,0.7 $.

The bootstrap variance estimator for $ \hat{F}_{np}(t) $ and $ \hat{F}_{p}(t) $ performed well (SER near 1) with all validation study sample sizes in most scenarios. Similarly, coverage for $ \hat{F}_{np}(t) $ and $ \hat{F}_{p}(t) $ was near the nominal value for all scenarios considered.

When validation sample size * m * was varied between 50 and 5,000, performance of the estimators varied by scenario. In scenarios A through D, absolute bias and RMSE were smaller for both $ \hat{F}_{np}(t) $ and $ \hat{F}_{p}(t) $ than for the naive estimator at all validation study sample sizes over 50 (Fig. 2). In Scenario E, where both missed events and false positive events occurred, $ \hat{F}_{np}(t) $ had greater bias than the naive estimator for all * m < * 1,000 and larger RMSE for nearly all values of * m * considered. $ \hat{F}_{p}(t) $ had smaller bias than the naive estimator for all values of * m * but greater RMSE than the naive estimator for * m < * 500. In Scenario F, where missed events, delayed event detection, and false positive events occurred, $ \hat{F}_{np}(t) $ had again larger bias than the naive estimator when * m < * 500 and larger RMSE than the naive estimator for nearly all values of * m * considered. In this scenario, $ \hat{F}_{p}(t) $ had little bias under all values of * m * and smaller RMSE than the naive estimator when * m≥ * 500.

Results were similar when the shape hazard function for the event of interest in the validation study differed from that in the main study (Fig. S6). As expected, the proposed approaches did not perform well when the gold standard validation data were themselves mismeasured (Fig. S7).

## 5. APPLICATION

In this section, we consider and account for outcome measurement error that could be induced by incomplete and/or incorrect linkage to death registries when estimating mortality risk among people entering care for HIV in the University of North Carolina Center for AIDS Research HIV Clinical Cohort between 2001 and 2022. Because gold standard data linkage is available in addition to an error-prone data linkage, we benchmark our approach against the gold standard approach to evaluate its performance in this setting.

### 5.1. Application methods

Participants were followed from the date of entry into HIV care at UNC on or after January 1, 2001 until death, administrative censoring on 31 December 2022, or 10 yr after entry into care. Gold standard death dates were available for the entire study cohort (*n* = 3,764) through a rigorous linkage and adjudication process administered by the UNC CFAR HIV Clinical Cohort. In a “gold-standard” analysis, we applied the Kaplan-Meier estimator to this dataset to compute the 10-yr risk of death.

To examine the performance of the estimator to account for outcome measurement error in this context, we created a second set of death dates for participants in the cohort by performing an exact match to the North Carolina death registry on first name, last name, and date of birth to produce an error-prone death date. In a “naive” analysis, we applied the Kaplan-Meier estimator to these error-prone death dates to compute the naive 10-yr risk of death.

Next, we randomly sampled 376 cohort members (10%) into a hypothetical internal validation study. We assumed that this error-prone date was available for the full cohort and the gold-standard death dates were available for only this sample of 376 participants. Using this validation study, we applied both parametric and nonparametric forms of the estimator to account for measurement error in the risk function over 10 yr.

To compute the nonparametric estimator of the risk function $ \hat{F}_{np}(t) $, $ \hat{a}(t)_{np} $ and $ \hat{b}(t)_{np} $ were recomputed at each event time in the validation data. To compute the parametric estimator $ \hat{F}_{p}(t) $, $ \hat{a}(t)_{p} $ and $ \hat{b}(t)_{p} $ were recomputed at each event time using the $ \hat{\lambda}_{fp} $, $ \hat{\lambda}_{d} $, and $ \hat{\theta} $ estimated in the full validation data. Estimated risk functions, 10-yr mortality risks, standard errors, and 95% CIs were compared between the approaches. In addition, pseudo root mean squared error was computed as the square root of the sum of the squared difference between the estimate from each method and the gold-standard estimate and the squared standard error for that approach. All analyses were performed using R 4.4.1.

### 5.2. Application results

Overall, 3,764 participants entered HIV care at UNC between 2001 and 2022. These participants were predominantly male and between 25 and 44 yr of age. Most study participants were Black (58%); 9% were Hispanic (Table S2). Participant characteristics in the hypothetical validation study were similar to those in the main study.

**Fig. 3.**
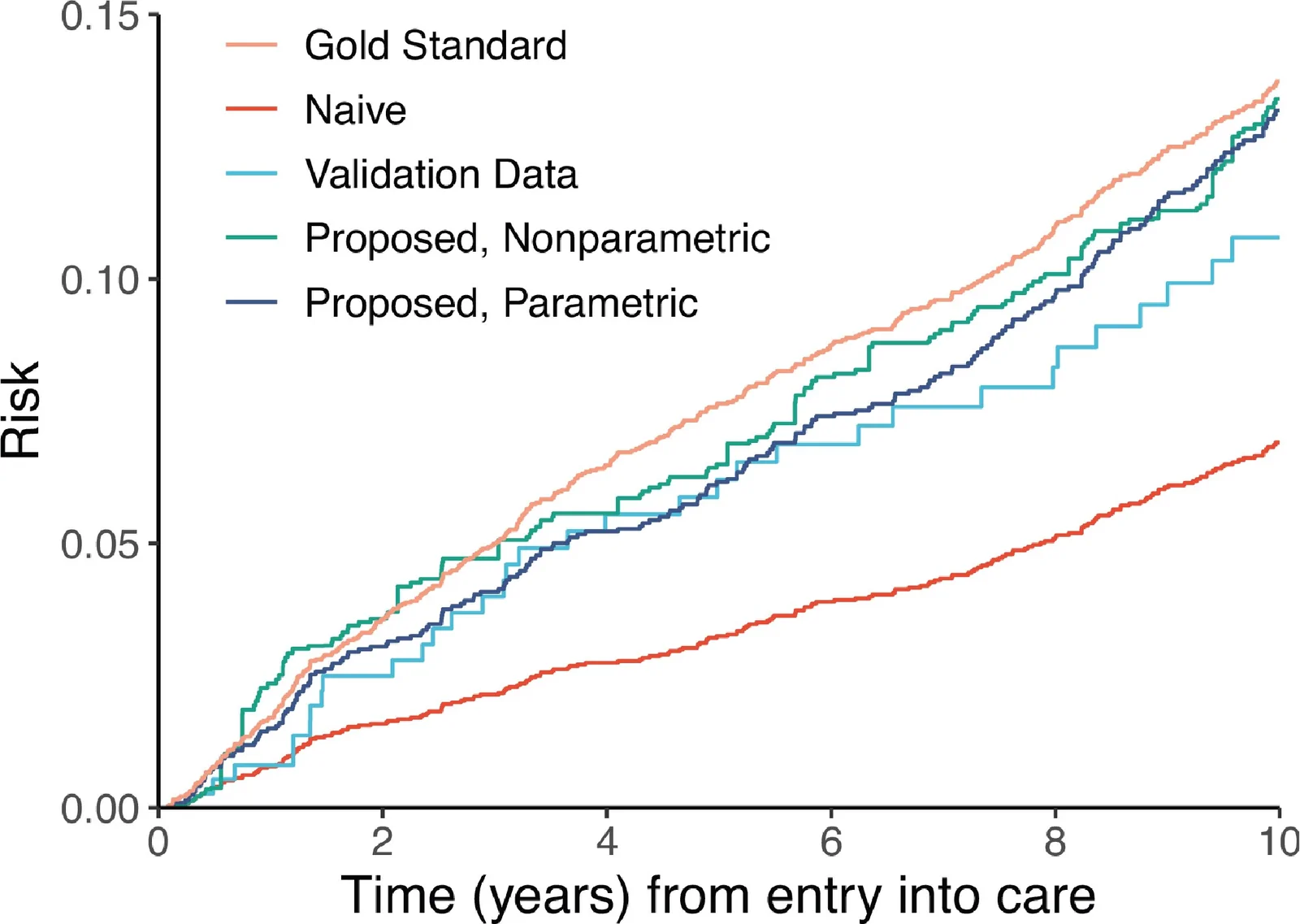
Mortality risk in the UNC CFAR Clinical HIV Cohort estimated using the gold standard analysis, the naive analysis, the validation study alone, and 2 analyses accounting for outcome measurement error.

Of the 3,764 participants, 432 deaths were recorded using the gold standard outcome measure, for a gold-standard 10-yr risk of 13.7% (95% CI: 12.5, 15.0) (Table 2). Using the error-prone exact linkage algorithm, 214 deaths were identified, for a naive 10-yr risk of 6.9% (95% CI: 6.0, 7.8). In the validation sample, 17 of 33 deaths (51.5%) were detected by the error-prone algorithm (16 “on-time” and 1 detected early) and no non-events were incorrectly classified as events. Accordingly, $ \theta\,{=}\,0.48 $, $ \lambda_{fp}=0.0004 $, and $ \lambda_{D}=0 $. Using data from the validation study alone, the estimated 10-yr risk was 10.8% (95% CI: 7.3, 14.3). After accounting for measurement error, estimated risks in the complete cohort were 13.4% (95% CI: 7.2. 19.7) and 13.2% (95% CI: 7.6, 18.8) for nonparametric and parametric estimators, respectively (Fig. 3 and Fig. S8). Pseudo root mean squared error was smaller for the parametric estimator than for the nonparametric estimator, and smaller for both estimators that accounted for the outcome measurement error than for estimators based on the naive outcome measurement or the validation study only.

**Table 2.** Estimated 10-yr mortality under 5 estimators among 3,764 people entering care for HIV in the UNC CFAR HIV Clinical Cohort, 2001–2022.

Analysis	Estimator	10-yr risk	Standard error	95% CI	*pseudo*-RMSE
Gold standard	$ \hat{F}(t) $	13.7	0.6	12.5, 15.0	Ref
Naive	$ \hat{F}_{W}(t) $	6.9	0.4	6.0, 7.8	6.8
Validation	$ \hat{F}_{T_{V}}(t) $	10.8	1.8	7.3, 14.3	3.4
Nonparametric	$ \hat{F}_{np}(t) $	13.4	3.2	7.2, 19.7	3.2
Parametric	$ \hat{F}_{p}(t) $	13.2	2.9	7.6, 18.8	2.9

## 6. DISCUSSION

This paper described parametric and nonparametric estimators to account for outcome measurement error in estimated risk functions. Here, the specific setting was outcome measurement error caused by incomplete and imperfect data linkage, but the proposed methods would be applicable to outcome measurement error caused by other mechanisms. The proposed approaches handle error in the measurement of the event times due to missed events, events detected late, and false positive events (including events detected prior to their true event times). Unlike traditional tabular methods for addressing outcome measurement error at a single timepoint [eg the Rogan Gladen estimator (Rogan and Gladen 1978)], the proposed estimators account for measurement error in the event times even when such error does not corrupt the event indicator. Therefore, these estimators can be used to account for mismeasurement of event times as well as misclassification of the event indicator.

In simulations, scenarios were examined with no measurement error and with false positives, missed events and/or delayed event detection. In all scenarios considered, simulations illustrated that—even when the absolute risk in the validation data did not match the risk in the main study—the proposed estimators yielded results with little bias and lower root mean squared error than a naive approach that did not account for measurement error. The performance of the estimators was tied to the size of the validation study when multiple types of measurement error were present (eg false positive events and missed events); in these settings, the proposed estimators only outperformed the naive estimator in terms of mean squared error when large validation datasets were used. As expected, the parametric estimator was more precise than the nonparametric estimator, and the nonparametric bootstrap variance estimator performed well. In settings with both missed and false positive events, the naive estimator outperformed the nonparametric estimator in terms of bias when the validation study had fewer than 1,000 participants and in terms of root mean squared error when the validation study had under 5,000 participants. Therefore, the nonparametric estimator, which allows maximum flexibility in the misclassification parameters, suffers in terms of precision and finite sample bias at realistic sample sizes. However, the parametric estimator performed well in terms of both bias and root mean squared error even when the validation sample was small.

In the application, the error-prone exact linkage algorithm suffered from severe underascertainment of deaths, resulting in an estimate of risk roughly half that of the true 10-yr mortality risk in this population. Because the application relied on state vital records, such underascertainment could be due to differences in the matching fields between the study data and the state registry or deaths occurring out of state and therefore missed by the state registry. Limiting the analysis to a 10% validation sample produced results closer to the truth but with poor precision. Applying the parametric and nonparametric approaches to account for outcome measurement error yielded results near the gold-standard results but less precise. An evaluation of the bias-precision tradeoff via comparison of pseudo root mean squared error nevertheless favored accounting for the measurement error using either the parametric or nonparametric estimator in this example. However, as seen in the simulations, in settings with small validation data and complex outcome measurement error, the bias-precision tradeoff may begin to favor the naive estimator.

Both parametric and nonparametric estimators of the measurement error parameters were provided. When applied to estimate the risk function, these estimators will typically provide similar estimates at the end of follow-up, as seen in simulations and example. However, as illustrated by the example, the shape of the risk functions may differ because the parametric estimator assumes that the false positive rate $ \lambda_{fp} $, event detection rate $ \lambda_{d} $, the probability of detecting an event on time * θ * are constant over time. However, a piecewise version of the parametric estimator may be used to relax these assumptions. For this estimator, measurement error parameters $ \hat{a}_{p}(t) $ and $ \hat{b}_{p}(t) $ are replaced with $ \tilde{a}_{p}(t) $ and $ \tilde{b}_{p}(t) $, which allow $ \lambda_{fp} $, $ \lambda_{d} $, and * θ * to update over time, which allows more flexibility in the estimated risk function. Small validation datasets may lack the support needed to accurately capture changes in $ \lambda_{fp} $, $ \lambda_{d} $, and * θ * over time.

The simulation results in Section 4.3 illustrate the tradeoffs between maximum flexibility in capturing changes to these parameters over time (ie the nonparametric estimator) and maximum stability and efficiency of the estimator (ie the parametric estimator that assumes $ \lambda_{fp} $, $ \lambda_{d} $, and * θ * are constant over time). Other options include allowing $ \lambda_{fp} $, $ \lambda_{d} $, and * θ * to be flexible parametric functions are interesting areas of future research. Note that even when $ \lambda_{fp} $, $ \lambda_{d} $, and * θ * are allowed to vary over time, the parametric estimator still makes simplifying assumptions about the relationship between * T * and each observed event time to estimate * a(t) *. These simplifying assumptions could be relaxed with detailed information on the expected distribution of * T *, however such information is usually unavailable in settings with outcome measurement error. However, these assumptions about the distribution of * T * were violated in the simulations shown in Section 4 and the parametric estimator still performed well. In summary, the parametric estimator imposes constraints on the parameter space that do not fully capture the nature of the outcome measurement error. However, these constraints allow progress in settings where the nonparametric estimator fails due to inadequate validation data.

As seen in simulations, the approach performed well even when the gold standard absolute risk and shape of the hazard function in the validation sample differed substantially from the main study, illustrating that the proposed estimators provide accurate estimates of risk in the target population even when analysis of the validation sample alone would not. This property implies that many different types of validation study designs could be used to inform the proposed estimators, including those that rely on efficient non-representative samples from the target population (Holcroft and Spiegelman 1999) or external validation data. With the growth and increased accessibility of large administrative data sources (such as electronic health records and claims data), the ability to incorporate external validation data is an advantage of this approach. With greater shared use of data resources have emerged calls for publishing reports of validation studies and sharing validation data between groups (Ehrenstein et al. 2016; Lash and Olshan 2016). Moreover, the ability of the approach to incorporate expert knowledge in place of validation data broadens its utility to include sensitivity analyses in settings where validation data are not available or quantitative bias analyses in settings where knowledge about the misclassification parameters is limited. However, when appropriately sampled internal validation data are available, the proposed approach may be less efficient than traditional “missing data” approaches to handle outcome measurement error (Edwards et al. 2013; Shepherd and Shaw 2020) because the proposed approach does not utilize the gold standard outcomes directly.

In some settings, the validation data itself may contain mismeasured gold standard outcomes or the “external knowledge” used to parameterize the estimators may be incorrect (Cole et al. 2024). In these settings, the proposed estimators would not be expected to perform well. When error in validation data is likely or external knowledge is highly uncertain, the proposed approach may be better viewed as a sensitivity analysis or quantitative bias analysis and used to evaluate potential bias that may be present under plausible values of the misclassification parameters.

To aid in the application of these estimators to account for outcome measurement error in other settings, we have developed the “Handling error in event times”: heet package for R, which can be found at https://github.com/edwardsjk/heet. The package takes as inputs (i) the error-prone event times and indicators; and (ii) the true and error-prone event times and indicators from a validation study. Outputs include estimated risk at the end of follow-up, estimated standard error, and the risk function over the full follow up period.

## Supplementary Material

kxaf052_Supplementary_Data

## Software availability

Code for the simulation experiments example analysis are available at https://github.com/edwardsjk/risk_outcome_measerr.

An R package to implement the approach to account for measurment error in survival analysis described here is available on GitHub at https://github.com/edwardsjk/heet.
