# Supplementary material for: Risk functions with outcome measurement error
Source: Biostatistics. 2026 Jan 20;27(1):kxaf052. doi: 10.1093/biostatistics/kxaf052 (PMC12815892; doi:10.1093/biostatistics/kxaf052)
Supplement: kxaf052_Supplementary_Data [file kxaf052_supplementary_data.pdf]

Biostatistics (2025), **0**, 0, *pp.* 1–**??**
doi:10.1093/biostatistics/supplement

Risk functions with outcome misclassification: Supplementary Material

JESSIE K. EDWARDS*[1], STEPHEN R. COLE[1], PAUL N. ZIVICH[1], BENJAMIN ACKERMAN [2], SONIA NAPRAVNIK [3], HEATHER HENDERSON [3], TIMOTHY L. LASH[4], BONNIE E. SHOOK-SA[5]

[1] *Department of Epidemiology University of North Carolina at Chapel Hill, Chapel Hill, NC USA*
[2] *Johnson and Johnson, NJ, USA* [3] *School of Medicine University of North Carolina at Chapel Hill, Chapel Hill, NC USA* [4] *Department of Epidemiology Emory University, Atlanta, GA, USA*
[5] *Department of Biostatistics University of North Carolina at Chapel Hill, Chapel Hill, NC USA*

jessedwards@unc.edu

1. Derivation of estimator 3.1

Our quantity of interest is $F(t) = F_T(t) = P(T \leqslant t)$. Let $S_T(t) = 1 - F_T(t), F_W(t) = P(W \leqslant t), S_W(t) = 1 - F_W(t), a(t) = P(W \leqslant t|T \leqslant t)$, and $b(t) = P(W \leqslant t|T > t)$.

We can rewrite $F_W(t)$ as a function of $F(t), a(t)$, and $b(t)$,

$$F_W(t) = F_T(t)a(t) + S_T(t)b(t) \tag{1.1}$$

Rearranging,

$$F(t) = \frac{(F_W(t) - S(t)b(t))}{(a(t))}$$

*To whom correspondence should be addressed.

© The Author 2025. Published by Oxford University Press. All rights reserved. For permissions, please e-mail: journals.permissions@oup.com

Similarly,

$$S_W(t) = S(t)(1 - b(t)) + F(t)(1 - a(t))$$

Rearranging, we can say

$$S(t) = \frac{S_W(t) - F(t)[1 - a(t)]}{[1 - b(t)]}$$

Now we can substitute this expression for S(t) into 1.2, such that

$$F(t) = \frac{F_W(t) - \frac{[S_W(t) - F(t) + F(t)a(t)]}{[1 - b(t)]} b(t)}{a(t)}$$

Which simplifies to

$$\begin{aligned} F(t)a(t) &= F_W(t) - \frac{[S_W(t) - F(t) + F(t)a(t)]b(t)}{1 - b(t)} \\ &= \frac{F_W(t)[1 - b(t)] - [S_W(t) - F(t) + F(t)a(t)]b(t)}{1 - b(t)} \\ &= \frac{F_W(t) - F_W(t)b(t) - S_W(t)b(t) + F(t)b(t) - F(t)a(t)b(t)}{1 - b(t)} \end{aligned}$$

Which implies that

$$F(t)a(t)(1 - b(t)) = F_W(t) - F_W(t)b(t) - [1 - F_W(t)]b(t) + F(t)b(t) - F(t)a(t)b(t)$$

And thus,

$$F(t)a(t) - F(t)a(t)b(t) = F_W(t) - F_W(t)b(t) - [1 - F_W(t)]b(t) + F(t)b(t) - F(t)a(t)b(t)$$

Solving for $F(t)a(t)$ yields

$$\begin{aligned} F(t)a(t) &= F_W(t) - F_W(t)b(t) - b(t) + F_W(t)b(t) + F(t)b(t) \\ &= F_W(t) - b(t) + F(t)b(t) \end{aligned}$$

Therefore,

$$F(t)a(t) - F(t)b(t) = F_W(t) - b(t)$$

Which is equal to

$$F(t) = \frac{F_W(t) - b(t)}{a(t) - b(t)}$$

motivating estimator 3.1 in the main text.

2. Consistency of nonparametric estimators for $a(t)$ and $b(t)$

Recall that $a(t)$ is the probability of being classified as an event by time t, given that one truly had the event at or before t, defined as $a(t) = P(W \leqslant t | T \leqslant t)$. Our nonparametric estimator for $a(t)$ at each event time R_k is

$$\hat{a}_{np}(R_k) = \frac{\sum_{i=1}^{m} I(W_i^* \leqslant R_k, T_i^* \leqslant R_k)\delta_i \eta_i}{\sum_{i=1}^{m} I(T_i^* \leqslant R_k)\delta_i} \tag{2.2}$$

where m is the number of participants in the validation data.

If censoring is noninformative, the expected value of the estimator shown in Equation 2.2 is equal to

$$E\left[\frac{\sum_{i=1}^{m} I(W_i \leqslant R_k, T_i \leqslant R_k)}{\sum_{i=1}^{m} I(T_i \leqslant R_k)}\right] \tag{2.3}$$

because the probability of being censored at each timepoint is the same for events and non-events.

By the law of large numbers $\frac{\sum_{i=1}^{m} I(W_i \leqslant R_k, T_i \leqslant R_k)}{\sum_{i=1}^{m} I(T_i \leqslant R_k)}$ converges in probability to $\frac{P(W \leqslant R_k, T \leqslant R_k)}{P(T \leqslant R_k)}$ for all R_k.

By the laws of conditional probability, $\frac{P(W \leqslant R_k, T \leqslant R_k)}{P(T \leqslant R_k)} = P(W \leqslant R_k | T \leqslant R_k) = a(R_k)$.

Similar logic can be applied to show proof of consistency for $\hat{b}_{np}(R_k)$.

3. Approach to simulate outcome measurement error

To simulate "error-prone" event times and event indicators for each individual, we used the following algorithm:

1. Induce imperfect event detection (i.e., specify $\theta < 1$): for all true events $\delta = 1$, draw an intermediate value of the observed event indicator η_1 from a Bernoulli distribution with probability θ. Set an intermediate value of the observed event time W_1^* to T^* if $\delta = \eta_1$ and the first of the end of follow up τ and the individual's censoring time C if $\delta = 1$ and $\eta_1 = 0$.

2. Induce delayed event detections: Draw the time from event to event detection T_{delay} from an exponential distirbution determined by the event detection rate λ_d. Create an intermediate time variable W_2^* that is set to T^* if $\eta_1 = \delta$ and $\min(T^* + T_{delay}, \tau, C)$ if $\delta = 1$ and $\eta_1 = 0$. A second intermediate event indicator η_2 was set to 1 if $W_2^* < \min(\tau, C)$, and was otherwise set to η_1.

3. Induce false positives: Draw the time to a false positive event (T_{fp}) from an exponential distribution determined by the false positive event rate λ_{fp}. Create a third intermediate value of the observed time to event $W_3^* = \min(T_{fp}, \tau, C)$.

4. The final error prone time to event was $W^* = \min(W_3^*, W_2^*)$ and the error-prone event indicator η was set to 1 if $W^* < T^*$ and η_2 otherwise.

4. Supplemental results

Table 1. *Measurement error parameters for simulated scenarios A-F and types of participants from Figure 1 from the main text in the simulated population*

Scenario	θ	λ_{fp}	λ_D	Types of participants in Figure 1 from the main text
A	1	0	0	IDs 1 and 2
B	0.7	0	0	IDs 1, 2, and 3
C	0.7	0	0.3	IDs 1, 2, 3, and 6
D	1	0.1	0	IDs 1, 2, 4, and 5
E	0.7	0.1	0	IDs 1, 2, 3, 4, and 5
F	0.7	0.1	0.3	IDs 1, 2, 3, 4, 5, and 6

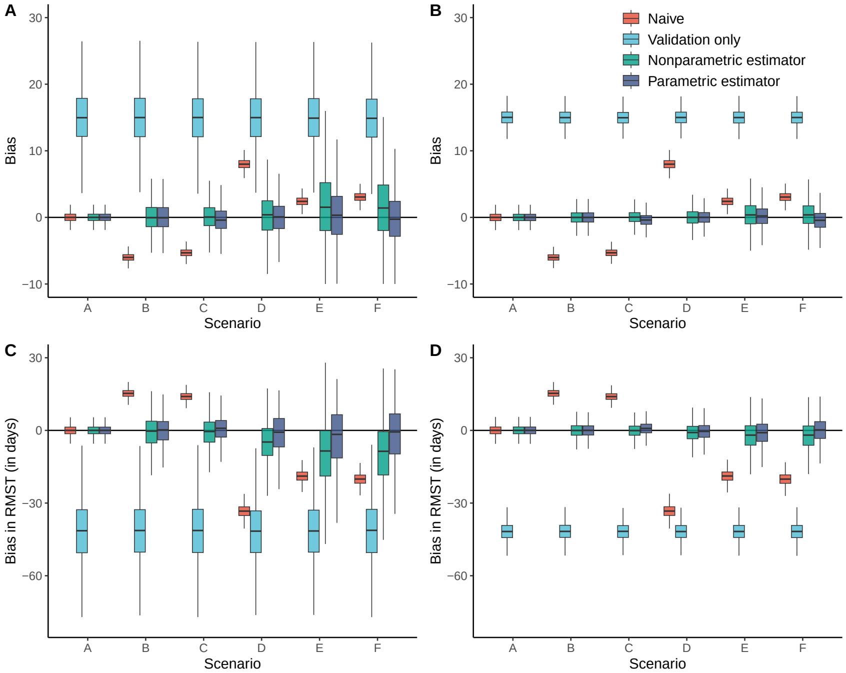

Fig. 1. Boxplot of bias in estimated risk at the end of follow up (panels A and B) and bias in the estimated restricted mean survival time (RMST) through the end of follow up (panels C and D) across 10,000 simulated cohorts of size $n = 5000$ with a validation sample size $m = 200$ (panels A and C) and $m = 2500$ (panels B and D) using 4 estimators of risk under 6 measurement error scenarios: A no measurement error; B $\lambda_{fp} = 0, \lambda_D = 0, \theta = 0.7$; C $\lambda_{fp} = 0; \lambda_D = 0.3; \theta = 0.7$; D $\lambda_{fp} = 0.1; \lambda_D = 0; \theta = 1$; E $\lambda_{fp} = 0.1; \lambda_D = 0; \theta = 0.7$; and F $\lambda_{fp} = 0.1; \lambda_D = 0.3; \theta = 0.7$.

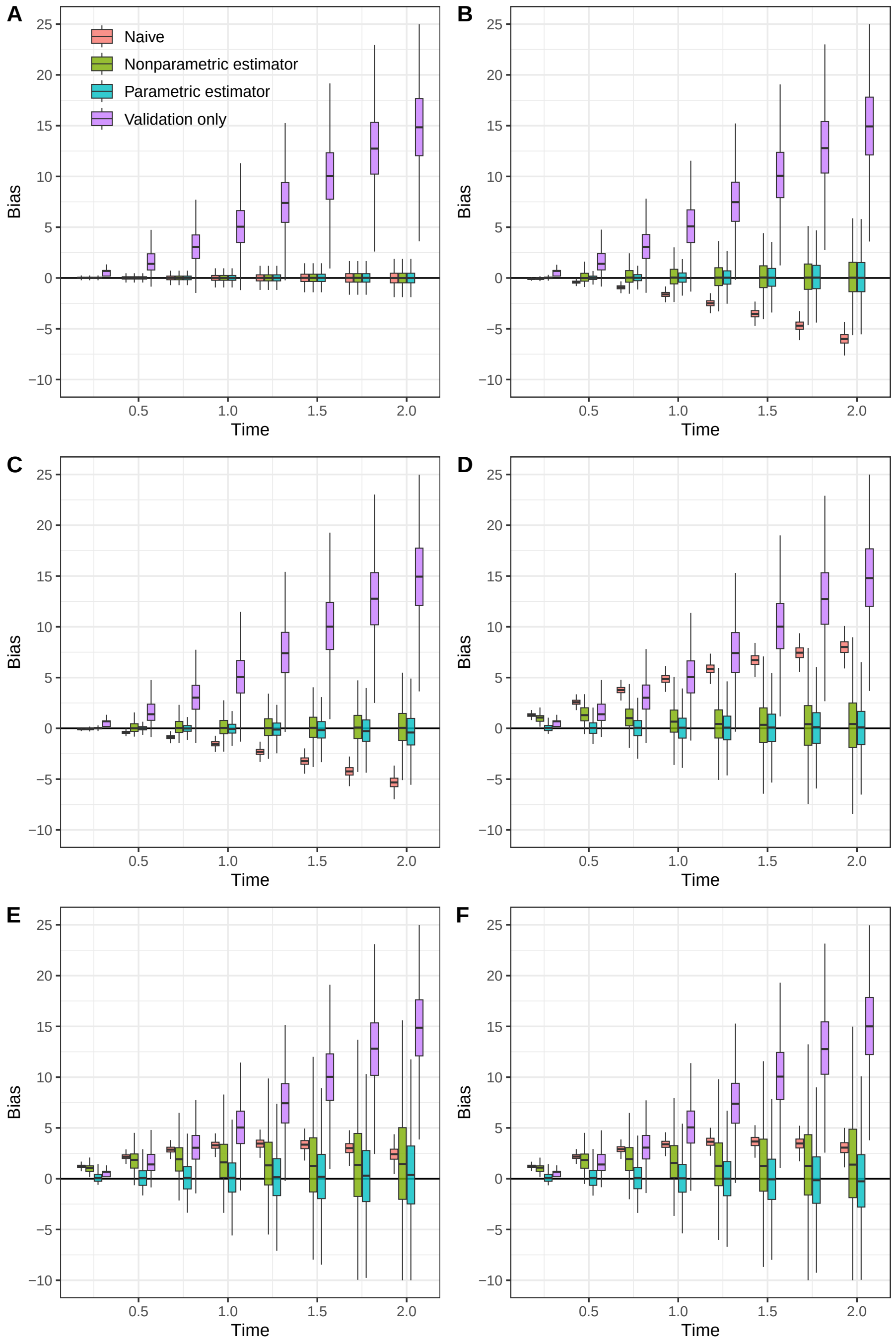

Fig. 2. Boxplot of bias in estimated risk at 8 timepoints across 10,000 simulated cohorts of size n=5000 with validation sample size $n_{val} = 200$ using 4 estimators of risk under 6 measurement error scenarios: A no measurement error; B $\lambda_{fp} = 0, \lambda_D = 0, \theta = 0.7$; C $\lambda_{fp} = 0; \lambda_D = 0.3; \theta = 0.7$; D $\lambda_{fp} = 0.1; \lambda_D = 0; \theta = 1$; E $\lambda_{fp} = 0.1; \lambda_D = 0; \theta = 0.7$; and F $\lambda_{fp} = 0.1; \lambda_D = 0.3; \theta = 0.7$.

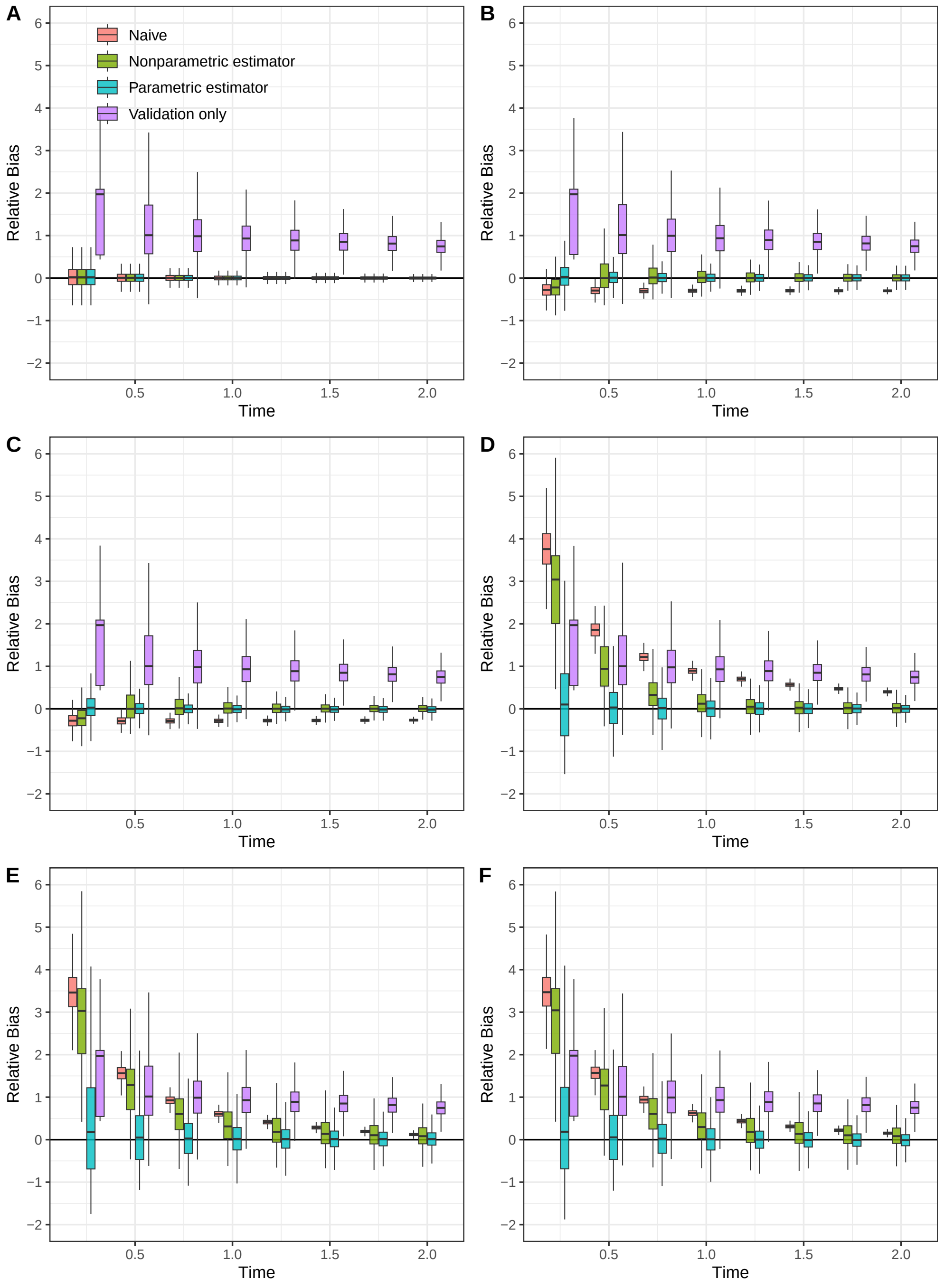

Fig. 3. Boxplot of relative bias in estimated risk at 8 timepoints across 10,000 simulated cohorts of size n=5000 with validation sample size $n_{val} = 200$ using 4 estimators of risk under 6 measurement error scenarios: A no measurement error; B $\lambda_{fp} = 0, \lambda_D = 0, \theta = 0.7$; C $\lambda_{fp} = 0; \lambda_D = 0.3; \theta = 0.7$; D $\lambda_{fp} = 0.1; \lambda_D = 0; \theta = 1$; E $\lambda_{fp} = 0.1; \lambda_D = 0; \theta = 0.7$; and F $\lambda_{fp} = 0.1; \lambda_D = 0.3; \theta = 0.7$.

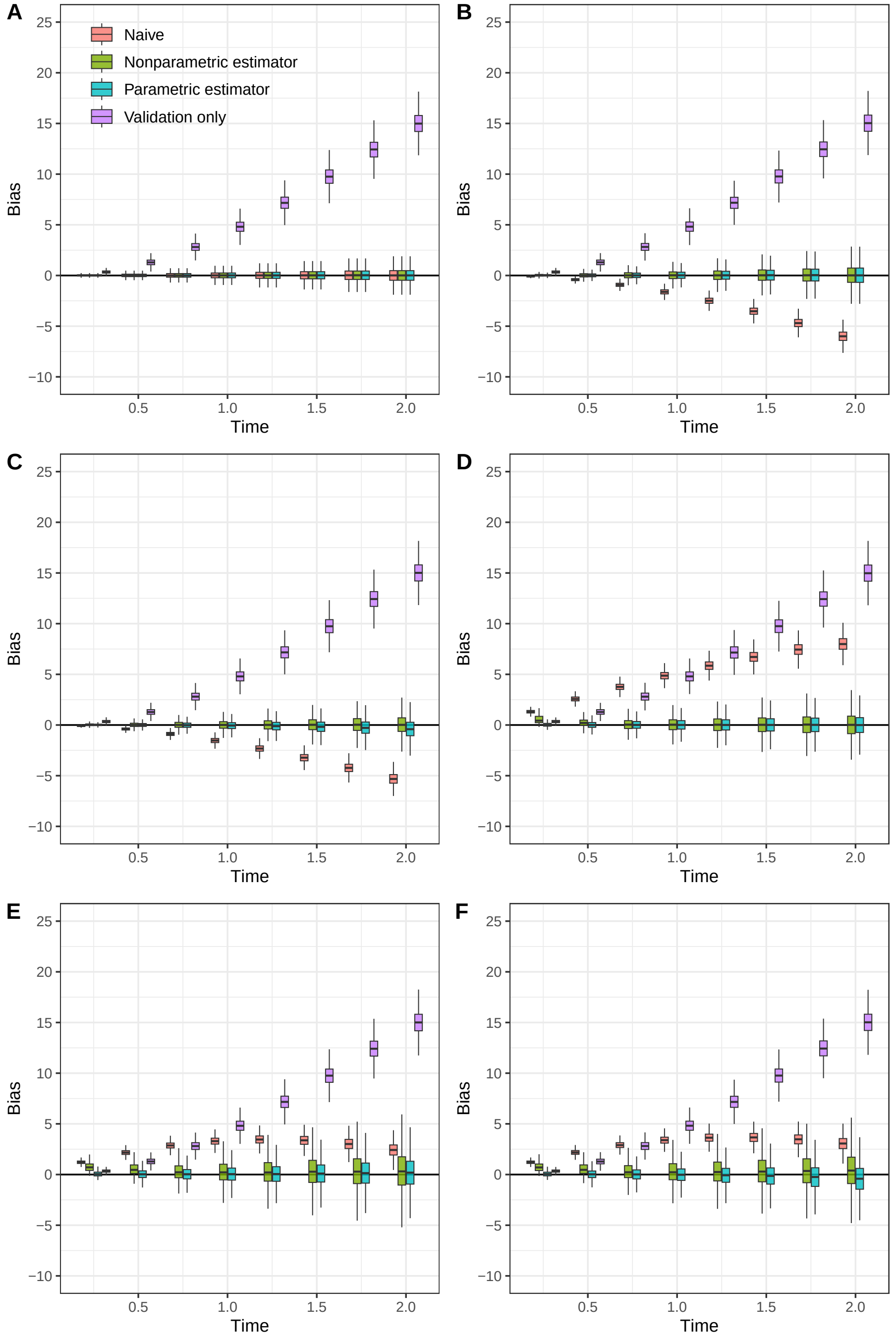

Fig. 4. Boxplot of bias in estimated risk at 8 timepoints across 10,000 simulated cohorts of size n=5000 with validation sample size $n_{val} = 2500$ using 4 estimators of risk under 6 measurement error scenarios: A no measurement error; B $\lambda_{fp} = 0, \lambda_D = 0, \theta = 0.7$; C $\lambda_{fp} = 0; \lambda_D = 0.3; \theta = 0.7$; D $\lambda_{fp} = 0.1; \lambda_D = 0; \theta = 1$; E $\lambda_{fp} = 0.1; \lambda_D = 0; \theta = 0.7$; and F $\lambda_{fp} = 0.1; \lambda_D = 0.3; \theta = 0.7$.

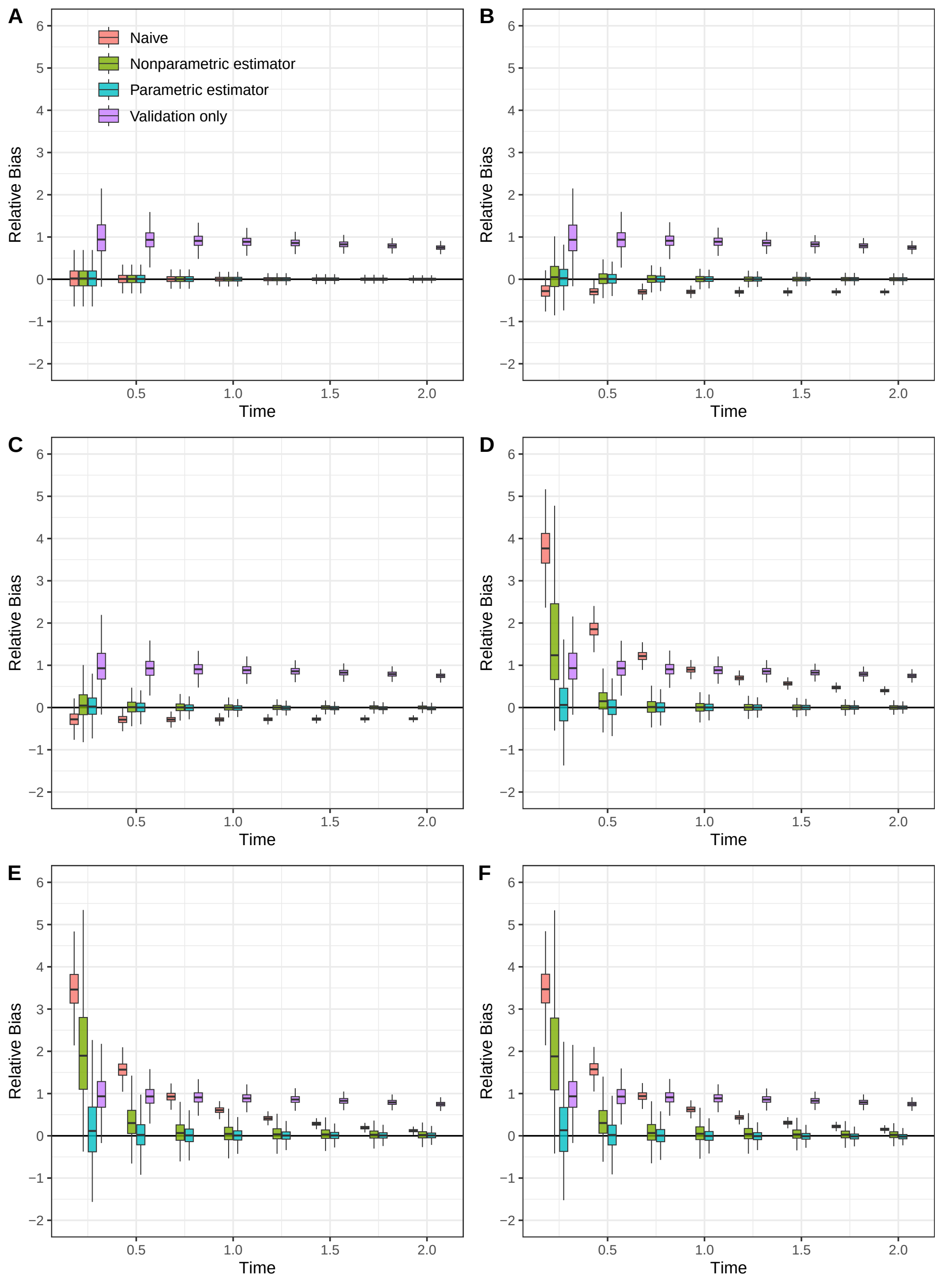

Fig. 5. Boxplot of relative bias in estimated risk at 8 timepoints across 10,000 simulated cohorts of size n=5000 with validation sample size $n_{val} = 2500$ using 4 estimators of risk under 6 measurement error scenarios: A no measurement error; B $\lambda_{fp} = 0, \lambda_D = 0, \theta = 0.7$; C $\lambda_{fp} = 0; \lambda_D = 0.3; \theta = 0.7$; D $\lambda_{fp} = 0.1; \lambda_D = 0; \theta = 1$; E $\lambda_{fp} = 0.1; \lambda_D = 0; \theta = 0.7$; and F $\lambda_{fp} = 0.1; \lambda_D = 0.3; \theta = 0.7$.

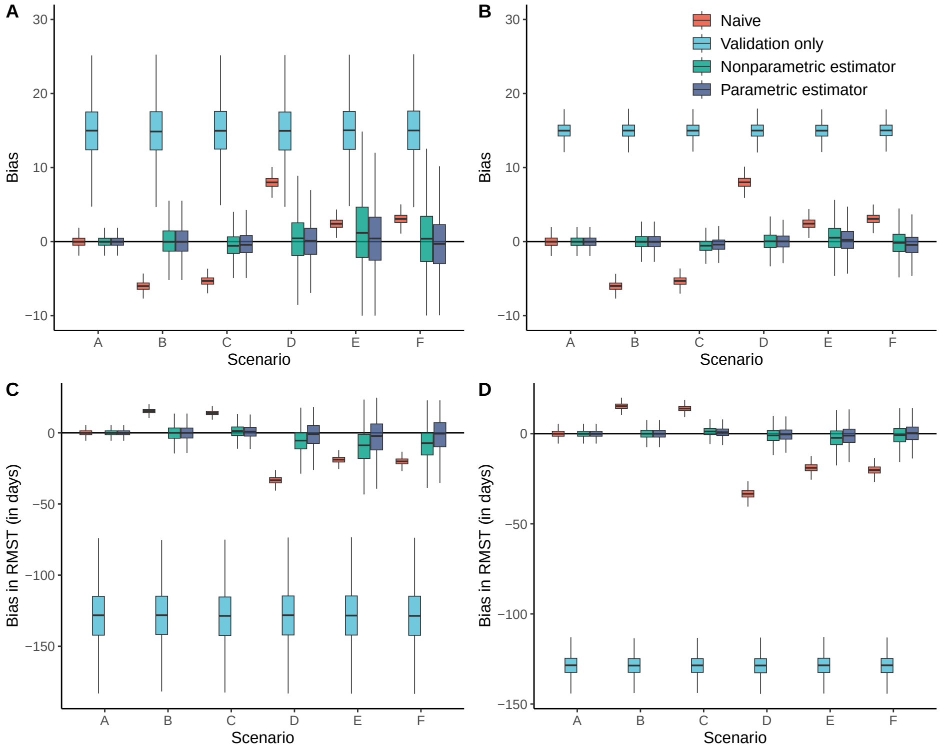

Fig. 6. Boxplot of bias in estimated risk at the end of follow up (panels A and B) and bias in the estimated restricted mean survival time through the end of follow up (panels C and D) when the validation study was generated with different shape and scale parameters across 10,000 simulated cohorts of size $n = 5000$ with a validation sample size $m = 200$ (panels A and C) and $m = 2500$ (panels B and D) using 4 estimators of risk under 6 measurement error scenarios: A no measurement error; B $\lambda_{fp} = 0, \lambda_D = 0, \theta = 0.7$; C $\lambda_{fp} = 0; \lambda_D = 0.3; \theta = 0.7$; D $\lambda_{fp} = 0.1; \lambda_D = 0; \theta = 1$; E $\lambda_{fp} = 0.1; \lambda_D = 0; \theta = 0.7$; and F $\lambda_{fp} = 0.1; \lambda_D = 0.3; \theta = 0.7$.

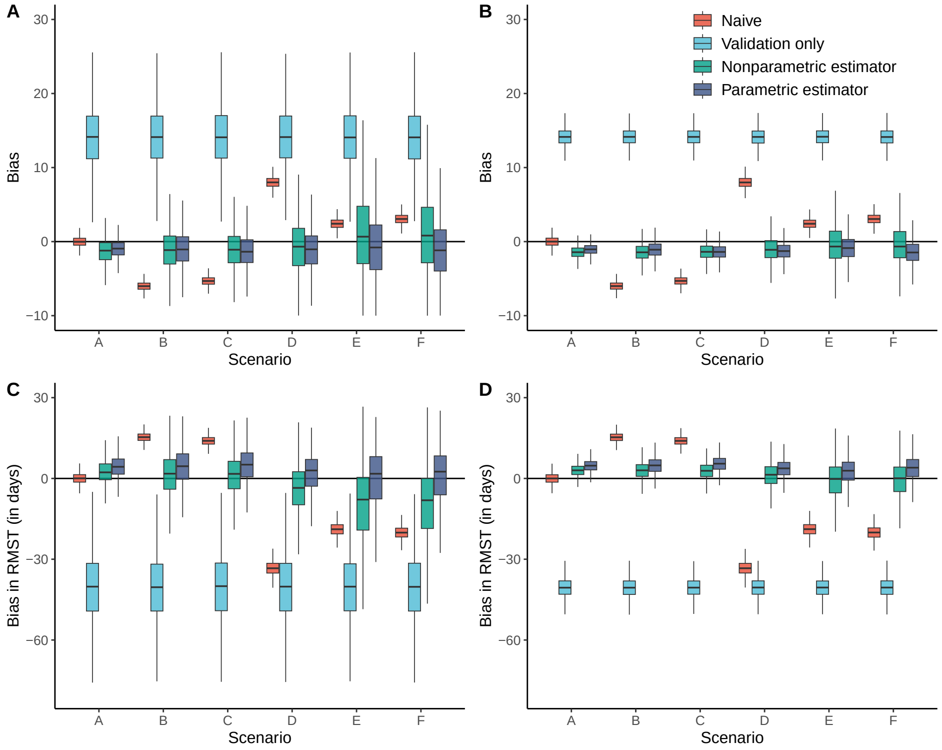

Fig. 7. Boxplot of bias in estimated risk at the end of follow up (panels A and B) and bias in the estimated restricted mean survival time through the end of follow up (panels C and D) when the gold standard measurements in the validation study where themselves subject to error with $\theta = 0.95$, $\lambda_{fp} = 0.005$, and $\lambda_d = 0.2$ across 10,000 simulated cohorts of size $n = 5000$ with a validation sample size $m = 200$ (panels A and C) and $m = 2500$ (panels B and D) using 4 estimators of risk under 6 measurement error scenarios: A no measurement error; B $\lambda_{fp} = 0, \lambda_D = 0, \theta = 0.7$; C $\lambda_{fp} = 0; \lambda_D = 0.3; \theta = 0.7$; D $\lambda_{fp} = 0.1; \lambda_D = 0; \theta = 1$; E $\lambda_{fp} = 0.1; \lambda_D = 0; \theta = 0.7$; and F $\lambda_{fp} = 0.1; \lambda_D = 0.3; \theta = 0.7$.

Table 2. *Characteristics of 3764 participants in the UNC CFAR HIV Clinical Cohort Study at entry into HIV care at UNC, 2001 - 2022*

Covariate	Overall (n = 3764) n	%	Hypothetical validation study (n = 376) n	%
Male gender	2731	73%	288	77%
Age				
<25	534	14%	44	12%
[25, 35)	1049	28%	111	30%
[35, 45)	1038	28%	100	27%
[45, 55)	783	21%	79	21%
55+	360	10%	42	11%
Race				
White	1116	30%	114	30%
Black	2208	59%	215	57%
Asian/Pacific Islander	348	9%	36	10%
American Indian	43	1%	7	2%
Unknown	49	1%	4	1%
Hispanic	325	9%	33	9%
Year of entry into care				
2001 - 2004	793	21%	75	20%
2005 - 2009	971	26%	88	23%
2010 - 2014	971	26%	101	27%
2015 - 2022	1029	27%	112	30%

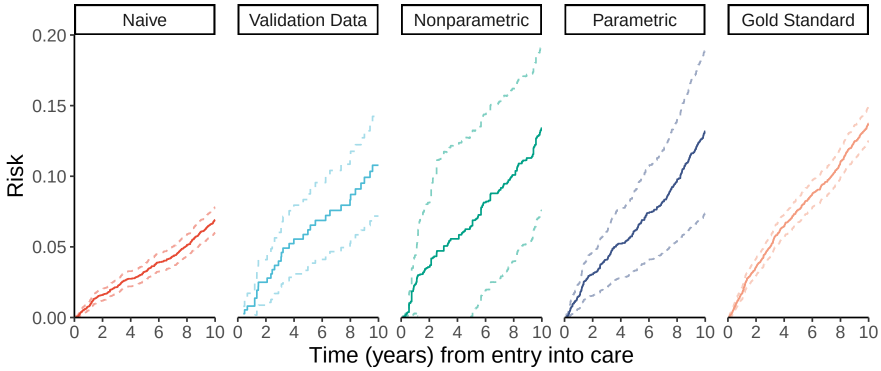

Fig. 8. Mortality risk in the UNC CFAR Clinical HIV Cohort estimated using the gold standard analysis, the naïve analysis, the validation study alone, and 2 analyses accounting for outcome measurement error, including 95% confidence intervals
